# Linear analysis of Atwood number effects on shear instability in the elastic–plastic solids

**DOI:** 10.1038/s41598-021-96738-1

**Published:** 2021-09-10

**Authors:** Xi Wang, Xiao-Mian Hu, Sheng-Tao Wang, Hao Pan, Jian-Wei Yin

**Affiliations:** 1 Institute of Applied Physics and Computational Mathematics, Beijing, 100094 People’s Republic of China; 2 Center for Applied Physics and Technology, Peking University, Beijing, 100871 People’s Republic of China

**Keywords:** Fluid dynamics, Surfaces, interfaces and thin films

## Abstract

The evolution of shear instability between elastic–plastic solid and ideal fluid which is concerned in oblique impact is studied by developing an approximate linear theoretical model. With the velocities expressed by the velocity potentials from the incompressible and irrotational continuity equations and the pressures obtained by integrating momentum equations with arbitrary densities, the motion equations of the interface amplitude are deduced by considering the continuity of normal velocities and the force equilibrium with the perfectly elastic–plastic properties of solid at interface. The completely analytical formulas of the growth rate and the amplitude evolution are achieved by solving the motion equations. Consistent results are performed by the model and 2D Lagrange simulations. The characteristics of the amplitude development and Atwood number effects on the growth are discussed. The growth of the amplitude is suppressed by elastic–plastic properties of solids in purely elastic stage or after elastic–plastic transition, and the amplitude oscillates if the interface is stable. The system varies from stable to unstable state as Atwood number decreasing. For large Atwood number, elastic–plastic properties play a dominant role on the interface evolution which may influence the formation of the wavy morphology of the interface while metallic plates are suffering obliquely impact.

## Introduction

Shear instability arises between two materials when there is a discontinuity of the tangential velocity at the perturbation interface. The stability problem for this configuration was first noticed by Lord Kelvin^1^ and Helmholtz^2^, and is popularly known as Kelvin–Helmholtz instability (KHI). The simplest form of the configuration treats two incompressible ideal fluid layers of constant densities *ρ*_1_ and *ρ*_2_ and constant tangential velocities *u*_1_ and *u*_2_. Linearization of the governing equations shows that the amplitude *ξ* of the interface develops exponentially, i.e. *ξ* ~ *ξ*_0_ exp(*σt*), in which the growth rate *σ* has the expression of {*ρ*_1_*ρ*_2_*k*^2^ [(*u*_1_ − *u*_2_)/(*ρ*_1_ + *ρ*_2_)]^2^}^1/2^^3^ where *k* is the wave number. The perturbation will grow if *u*_1_ ≠ *u*_2_. KHI in liquid or gas materials has been extensively treated in the literatures^1–14^. However, in contrast to the cases of fluids, KHI involving solids is historically less studied and far from deeply understood, despite the fact that KHI is a ubiquitous phenomenon in many engineering applications.

When a sliding detonation in high explosive spreads over a spherical steel capsule, the high-rate sliding motion of the explosive product induces a tangential velocity jump at the steel surface. The perturbation of 0.22 mm amplitude and 2.5 mm wavelength with plastic deformation was found in the surface fragment of the capsule because of the development of KHI along the steel surface^15^. The phenomenon of KHI was also detected at the interface between two closely packed metal plates (Al–Al and Al–Cu) when an oblique shock wave passed through. Bucking of the interface with periodic wavy morphology was formed^16^. Besides, KHI in solids is also of relevance to High Velocity Impact Welding (HVIW) which is a remarkable technique of joining a wide variety of both similar and dissimilar metals^17–22^. The principle of weld is by accelerating a flier workpiece with different energy sources (such as chemical explosive, repulsive magnetic field, laser-generated optical energy or electrically-driven rapid vaporization of a metallic conductor^23–25^) to a high velocity to obliquely impact a stationary workpiece. A successful weld is taken to be the emergence of wavy morphology at temperatures below the metal melting point with plastic deformation^26,27^. Many researchers have investigated the physical mechanism of formation of wavy interface^28–34^. Recently, the wavy pattern is considered to be the signature of a shear driven instability of a plastic material^34^. In addition, KHI is also a significant phenomenon in Inertial Confinement Fusion (ICF). Due to the impulsive nature of the laser-drivers in the experiments, KHI is less studied until an indirectly driven shock tube targets have been developed^35,36^. Both sides of an aluminum tracer foil are shocked by sustained counter-propagating shear flow to generate large velocities in the high-energy–density (HED) shear experiments on the National Ignition Facility (NIF). The formation of the wavy morphology induced by the development of KHI at metallic interface is a critical issue concerned by researchers. The wavy pattern performs the dynamic behaviors of metals which are obliquely impacted. The study of the physics of the evolution of the interface amplitude of KHI in solids is essentially valuable in practical engineering applications.

Differing from hydrodynamic instability in fluids, the physics of KHI in solids are primarily determined by the constitutive relations of the materials which present complex non-linear characteristics between the stress and the strain during deformations^37^. A representative feature of the non-linear behavior is the elastic–plastic (EP) transition which results in enhancing the difficulties of constructing the theory even in the linear growth stage of the instability. Drennov^38^ deduced the analytical theory by the classical normal mode methodology with the assumption of incompressible materials when an ideal fluid of finite thickness at a constant tangential velocity flowed over a half-space metallic medium with perfectly elastic properties. This case resembles the situation that one surface layer of metals has suffered the thermal softening. An analytical expression of a compatibility condition of the growth rate for the system was achieved after linearizing the governing equations. By considering the incapability of deriving analytical solution for elastoplastic medium, the stability boundary was coarsely estimated. Nassiri^34^ accomplished a more careful analysis of a system which was much closer to reality that two materials were treated as perfectly plastic behaviors. After applying the normal mode analysis to the linearized governing equations in the form of vorticity and stream function, the growth rates under different impact velocities were got by numerical solutions because of the impossibility to practice an analytical settlement. The normal mode analysis can describe the asymptotic behavior of the instability and the growth rates can be obtained. However, the characteristics of the development of the amplitude which may be affected by the properties of EP properties have not been mentioned, as well as extraordinarily complex mathematical problems are sufficiently involved in the normal mode analysis which lead to difficulties of achieving analytical formulas to deal with complex physical problems. Besides, previous theories were mainly restricted to KHI between two identical materials, though KHI usually emerges at the interface of materials with different densities. The effects of Atwood number (*A*_*T*_ = (*ρ*_*solid*_ − *ρ*_*fluid*_)/(*ρ*_*solid*_ + *ρ*_*fluid*_)) on the development of the amplitude have not been considered in the linear analysis.

The characteristic time of the velocity jump lasted for a short pulse of about 0.15 μs to adequately activate the increment of amplitude, after the metallic interface was loaded by an oblique shock wave^39^. The development of amplitude in the linear stage plays a significant role in such a short time, and it is advantageous to derive approximate analytical formulas for KHI in strength mediums to detect the physical insight of the development of the perturbed interface. The system of KHI between fluid and solid corresponds to the situations of explosive product sliding over metal surface^15^ or one of metals undergoing thermal softening after impact^38,39^. In present article, an approximate theoretical model for KHI between perfectly EP solid and ideal fluid with arbitrary densities is developed to achieve explicit analytical solutions to describe the evolution of the perturbation amplitude. The analytical expressions of growth rates and amplitude motions are theoretically deduced. The verification of the model is executed by numerical simulations and then, the effects of Atwood number on the development of KHI in the linear stage is studied by the model.

## Theoretical model formulation

### Fundamental equations

To develop the model, several assumptions are reasonably made. First, it is assumed that the main characteristics of the perturbation can be described in the two-dimensional (2D) and planar coordinates if the spanwise direction lengths of the plates perpendicular to the 2D section are long enough^16,38,39^. Second, on the condition that the scale of the wavelength *λ* is much smaller than that of material thickness *h*^25,39^, i.e. *kh* ≫ 1 where *k* = 2π/*λ*, it is reasonable to consider each material occupies infinite half space. Third, the plastic deformation is essentially an incompressible phenomenon with little change in densities and elastic strains are assumed to be far smaller than plastic ones. The assumption of constant densities is adopted. Forth, both of the materials are treated as uniform material properties. Fifth, the flow field is assumed to be irrotational. During the evolution of the interface, the perturbed velocity field is determined by conservation equations of the momentum with conservative or/and nonconservative forces acting on the perturbation interface. Although the nonconservative force arouses the rotation of the velocity field, the vorticity only affects a very small distance away from the interface^39^. With the assumption of irrotational flow field, not only the mathematical complexity is largely reduced to obtain some analytical formulas from physics insight, but also the relatively good accuracy of the model is guaranteed as shown by simulations as described in a later section.

**Figure 1:**
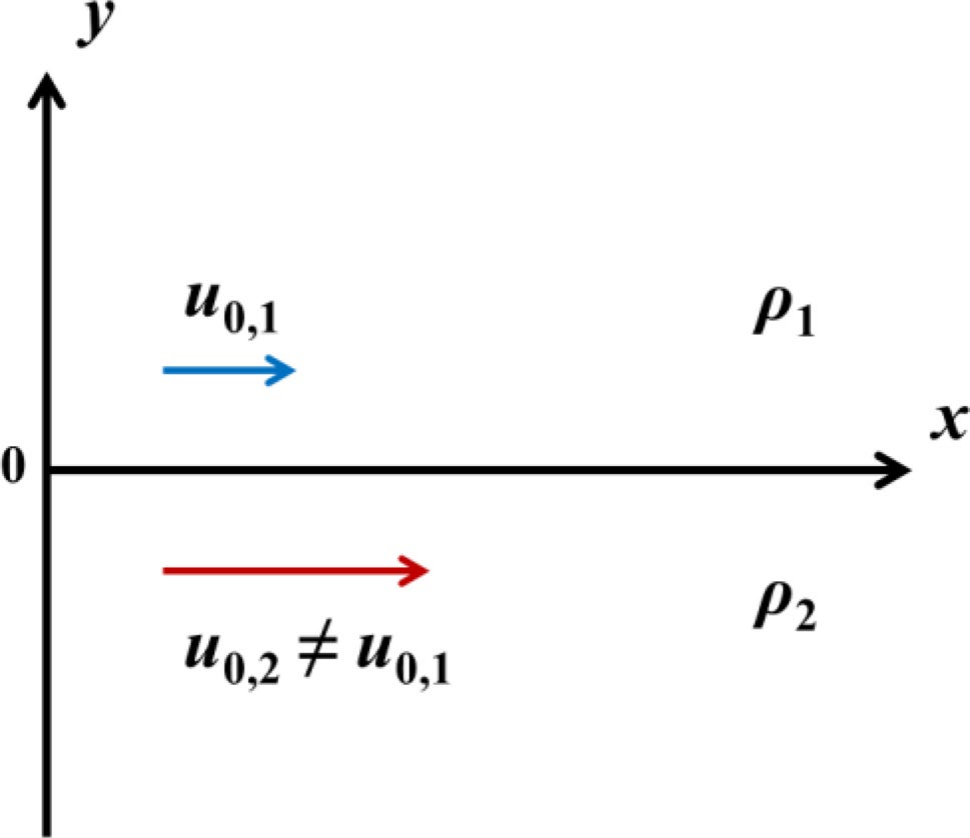
The schematic of the physical model.

Figure 1 shows the schematic of the KHI system in 2D Cartesian coordinates. The *x*-axis separates the two materials as an interface and the *y*-axis is oriented normal to the *x*-axis. Material 1 and 2 with constant density *ρ*_1_ and *ρ*_2_ occupy the upper (*y* > 0) and lower (*y* < 0) half-space respectively. Both solid and fluid have constant velocities with the values of *u*_0,1_ and *u*_0,2_ respectively in *x* direction. After disturbance, the interface deviates a infinitesimal distance from *y* = 0 to *y* = *η* (*x*, *t*) as a first-order term. The outward normal of the disturbed interface to the first-order term is

1 $$ {\varvec{n}} = - \frac{\partial \eta }{{\partial x}}{\varvec{i}} + {\varvec{j}}. $$

For an incompressible flow, the continuity equation is

2 $$ \nabla \cdot {\varvec{u}}_{i} = 0, $$

where *i* = 1 and *i* = 2 represents two materials respectively, ***u***_*i*_ characterizes the irrotational perturbed velocity of material *i*. If the materials are irrotational at the initial moment, the irrotational properties of the flow will be maintained. ***u***_*i*_ can be expressed by the perturbed velocity potential *ϕ*_*i*_

3 $$ {\varvec{u}}_{i} = \nabla \phi_{i} . $$

By combining Eq. (2), *ϕ*_*i*_ satisfies the Laplace equation. Considering each medium has a tangential velocity, a velocity potential can be written as

4 $$ \Phi_{i} = u_{0,i} x + \phi_{i} , $$

which still satisfies the Laplace equation.

Without conservative and nonconservative forces acting on the interface, the flow is governed by the momentum equation

5 $$ \rho_{i} \left[ {\frac{{\partial {\varvec{u}}_{i} }}{\partial t} + \left( {u_{0,i} \cdot \nabla } \right){\varvec{u}}_{i} } \right] = - \nabla p_{i} , $$

where *p*_*i*_ is pressure. Integrating Eq. (5) from *y* = 0 to the instantaneous interface *y* = *η* (*x*, *t*) in *y* direction and substituting Eq. (3), it has

6 $$ \begin{aligned} p_{i} & = \left. {p_{i} } \right|_{y = 0} - \rho_{i} \int_{0}^{\eta } {\frac{{\partial u_{i,y} }}{\partial t}} dy - \rho_{i} u_{0,i} \int_{0}^{\eta } {\frac{{\partial u_{i,y} }}{\partial x}dy} \\ & = \left. {p_{i} } \right|_{y = 0} - \rho_{i} \int_{0}^{\eta } {\frac{{\partial^{2} \phi_{i} }}{\partial t\partial y}dy} - \rho_{i} u_{0,i} \int_{0}^{\eta } {\frac{{\partial^{2} \phi_{i} }}{\partial x\partial y}dy} \\ & = \left. {p_{i} } \right|_{y = 0} - \rho_{i} \frac{{\partial \phi_{i} }}{\partial t} + \rho_{i} \left. {\frac{{\partial \phi_{i} }}{\partial t}} \right|_{y = 0} - \rho_{i} u_{0,i} \frac{{\partial \phi_{i} }}{\partial x} + \rho_{i} u_{0,i} \left. {\frac{{\partial \phi_{i} }}{\partial x}} \right|_{y = 0} \\ & = - \left( {\rho_{i} \frac{{\partial \phi_{i} }}{\partial t} + \rho_{i} u_{0,i} \frac{{\partial \phi_{i} }}{\partial x} + C_{i} } \right), \\ \end{aligned} $$

where *u*_*i*,*y*_ is the *y* component of the perturbed velocity. Before the interface is perturbed, we have *y* = *η* (*x*, *t*) = 0 in equilibrium, then *C*_1_ = *C*_2_.

After the interface quasi-statically departs an infinitesimal distance from *y* = 0 at a certain time named *t* = 0, the interface has the cosinusoidal form of *ξ*_0_cos*kx* where *ξ*_0_ is the perturbation amplitude at *t* = 0. Because of the velocity potential in Eq. (4) depending upon *x*, the interface can be expressed by

7 $$ \eta (x,t) = \xi \left( t \right)e^{ikx} , $$

for the mathematical convenience where *ξ*(*t*) is the perturbation amplitude and the potential of the perturbed velocity is

8 $$ \phi_{i} = A_{i} \left( t \right)e^{ \mp ky} e^{ikx} . $$

The negative and positive signs correspond to the conditions which the velocities vanish when the distance from the interface goes to positive and negative infinity respectively.

In the process of evolution, the continuity of the normal components of the velocities at the interface is required. The kinematic conditions with the velocity potential in Eq. (4) and the interface normal in Eq. (1) are

9 $$ \frac{\partial \eta }{{\partial t}} = \nabla \Phi_{1} \cdot {\varvec{n}} = \nabla \Phi_{2} \cdot {\varvec{n}}. $$

After neglecting the high-order terms, the velocity continuity in the normal direction is

10 $$ \frac{\partial \eta }{{\partial t}} = - u_{0,1} \frac{\partial \eta }{{\partial x}} + \frac{{\partial \phi_{1} }}{\partial y} = - u_{0,2} \frac{\partial \eta }{{\partial x}} + \frac{{\partial \phi_{2} }}{\partial y}. $$

Substituting Eq. (8) into Eq. (10), we can express *A*_*i*_(*t*) in terms of *ξ*(*t*)

11 $$ \dot{\xi }(t) = - kA_{1} (t) = kA_{2} (t) - iku_{0} \xi \left( t \right), $$

where the dot above *ξ*(*t*) means taking the derivative of *ξ*(*t*). It needs to be explained that the 2D coordinates system is fixed to the material 1 while deriving Eq. (11), i.e. *u*_0,1_ = 0 and *u*_0,2_ = *u*_0_. The reason is that the key of producing KHI is the relative discrepancy between the tangential velocities at perturbation interface but not the absolute values of tangential velocities. Therefore, *u*_0_ is the velocity jump in *x* direction at the interface.

Furthermore, the force equilibrium at the interface to the first order approximation is also required

12 $$ p_{1} - p_{2} + \sum\limits_{j} {F_{y}^{\left( j \right)} } = 0, $$

where *F*_*y*_^(*j*)^ denotes forces per unitary surface acting on both sides of the interface in the vertical direction. *F*_*y*_^(*j*)^ can be surface tension, viscosity, elasticity, plasticity or nonconservative force. In the system of KHI, the EP solid and ideal fluid are placed beyond and beneath the *x*-axis respectively, and the force due to EP properties of the solid acts on the perturbed interface. Thus, Eq. (12) becomes

13 $$ p_{1} - p_{2} - S_{1,yy}^{{\left( {ep} \right)}} = 0\;, $$

where *S*_1,*yy*_^(*ep*)^ represents the vertical component of the deviatoric stress for solid in elastic or plastic state.

The force equilibrium is first applied to the perfectly elastic solid whose constitutive relation has the linear form, i.e. Hookean solid^40^

14a $$ \dot{S}_{1,ij} = 2G_{1} D_{1,ij} , $$

14b $$ D_{1,ij} = \frac{1}{2}\left( {\frac{{\partial u_{1,i} }}{{\partial x_{j} }} + \frac{{\partial u_{1,j} }}{{\partial x_{i} }}} \right), $$

where *G*_1_ is the shear modulus and *D*_1,*ij*_ is the strain rate tensor. By combining Eqs. (3) and (8), the strain rate tensor becomes

15 $$ D_{1,ij} = A_{1} \left( t \right)e^{ikx - ky} k^{2} \left[ {\begin{array}{*{20}c} { - 1} & { - i} \\ { - i} & 1 \\ \end{array} } \right]. $$

Then, combining Eq. (11) and integrating Eq. (14a) from the initial perturbed interface *η*(*x*, 0) = *ξ*_0_*e*^*ikx*^, the stress tensor *S*_1,*ij*_ can be obtained

16 $$ S_{1,ij} = - 2G_{1} k\left( {\xi - \xi_{0} } \right)e^{ikx - ky} \left[ {\begin{array}{*{20}c} { - 1} & { - i} \\ { - i} & 1 \\ \end{array} } \right]. $$

Thus, the vertical component of the deviatoric stress in the elastic regime is

17 $$ S_{1,yy}^{{\left( {ep} \right)}} = - 2G_{1} k\left( {\xi - \xi_{0} } \right)e^{ikx - ky} . $$

Substituting Eqs. (6) and (17) into Eq. (13), we have the motion equation of the perturbation amplitude in perfectly elastic solid

18 $$ \ddot{\xi } + \frac{{2\rho_{2} u_{0} ki}}{{\rho_{1} + \rho_{2} }}\dot{\xi } - \frac{{\rho_{2} u_{0}^{2} k^{2} }}{{\rho_{1} + \rho_{2} }}\xi = - \frac{{2G_{1} k^{2} }}{{\rho_{1} + \rho_{2} }}\left( {\xi - \xi_{0} } \right). $$

If the effective stress

19 $$ \overline{\sigma } = \sqrt {\tfrac{3}{2}S_{1,ij} S_{1,ij} } , $$

arrives at *Y* which is the yield stress of the solid, the solid will mechanically transit from the elastic regime into the plastic regime. By substituting Eq. (16), it has

20 $$ \overline{\sigma }^{2} = \frac{3}{2}S_{1,ij} S_{1,ij} = 12e^{ - 2ky} G_{1}^{2} k^{2} \left( {\xi - \xi_{0} } \right)^{2} . $$

Here, only the real components of the stress tensor are kept during deriving the effective stress due to express the cosinusoidal form of the interface as Eq. (7) for the mathematical convenience. Considering the plastic deformation only occurs at a small layer from the interface^39^, we take *y* = *k*^-1^ in Eq. (20) to get a better fit with numerical simulation results and find the perturbation amplitude *ξ*_*p*_ at when the elastic–plastic transition takes place

21 $$ \xi_{p} - \xi_{0} \approx \frac{eY}{{2\sqrt 3 G_{1} k}}. $$

Then, the vertical component of the deviatoric stress in the plastic regime becomes

22 $$ S_{1,yy}^{{\left( {ep} \right)}} = - \frac{eY}{{\sqrt 3 }}\;e^{ikx - ky} . $$

Combining Eq. (18), the motion equation of the amplitude of the perturbation interface in KHI between EP solid and ideal fluid can be achieved

23 $$ \ddot{\xi } + \frac{{2\rho_{2} u_{0} ki}}{{\rho_{1} + \rho_{2} }}\dot{\xi } - \frac{{\rho_{2} u_{0}^{2} k^{2} }}{{\rho_{1} + \rho_{2} }}\xi = - \left\{ \begin{gathered} \frac{{2G_{1} k^{2} }}{{\rho_{1} + \rho_{2} }}\left( {\xi - \xi_{0} } \right)\;,\quad \xi \le \xi_{p} \hfill \\ \frac{e}{\sqrt 3 }\frac{kY}{{\rho_{1} + \rho_{2} }}\;.\quad \quad \;\;\xi > \xi_{p} \hfill \\ \end{gathered} \right. $$

For two ideal fluids, the above equation degrades into

24 $$ \ddot{\xi } + \frac{{2\rho_{2} u_{0} ki}}{{\rho_{1} + \rho_{2} }}\dot{\xi } - \frac{{\rho_{2} u_{0}^{2} k^{2} }}{{\rho_{1} + \rho_{2} }}\xi = 0, $$

which is the same as previous result^6^.

It is convenient to write Eq. (23) as

25 $$ \ddot{\xi } + 2M_{1} i\;\dot{\xi } = \left\{ \begin{gathered} \Lambda \xi_{0} - \left( {\Lambda - M_{2} } \right)\xi \;,\quad \xi \le \xi_{p} \hfill \\ M_{2} \xi - {\rm X}\;,\quad \quad \quad \quad \;\,\xi > \xi_{p} \hfill \\ \end{gathered} \right. $$

with the definitions of

26 $$ M_{1} = \frac{{\rho_{2} u_{0} k}}{{\rho_{1} + \rho_{2} }},M_{2} = \frac{{\rho_{2} u_{0}^{2} k^{2} }}{{\rho_{1} + \rho_{2} }},\Lambda = \frac{{2G_{1} k^{2} }}{{\rho_{1} + \rho_{2} }},{\rm X} = \frac{e}{\sqrt 3 }\frac{kY}{{\rho_{1} + \rho_{2} }}. $$

The corresponding initial conditions are

27 $$ \xi \left( 0 \right) = \xi_{0} ,\quad \quad \dot{\xi }\left( 0 \right) = 0. $$

### Growth rate

The eigenvalue function of Eq. (25) is the dispersion relation

28 $$ n^{2} + 2M_{1} i\;n = \left\{ \begin{gathered} - \left( {\Lambda - M_{2} } \right),\quad \xi \le \xi_{p} \hfill \\ M_{2} ,\quad \quad \quad \quad \xi > \xi_{p} \hfill \\ \end{gathered} \right. $$

where *n* is the eigenvalue. Equation (28) is a quadratic polynomial formula that has complex solutions. In the elastic branch, the solutions are

29 $$ \begin{aligned} n_{e} & = - M_{1} i \pm \sqrt { - M_{1}^{2} - \left( {\Lambda - M_{2} } \right)} \\ & = - \frac{{\rho_{2} ku_{0} }}{{\rho_{1} + \rho_{2} }}i \pm \frac{{k\sqrt {\rho_{1} \rho_{2} u_{0}^{2} - 2G_{1} \left( {\rho_{1} + \rho_{2} } \right)} }}{{\rho_{1} + \rho_{2} }}, \\ \end{aligned} $$

where the index *e* denotes the elastic state of the solid. Equation (29) can be degenerated to the case of two ideal fluids which is the same as Ref.^6^. *n*_*e*_ may contain real and imaginary parts. If the following condition in Eq. (30) is satisfied

30 $$ u_{0} > u_{0,cr} = \sqrt {2G_{1} \frac{{\rho_{1} + \rho_{2} }}{{\rho_{1} \rho_{2} }}} = \sqrt {\frac{{4G_{1} }}{{\rho_{1} \left( {1 - A_{T} } \right)}}} , $$

where *u*_0,*cr*_ is the critical value of the tangential velocity jump, the formula in the radical sign in Eq. (29) is positive and *n*_*e*_ possesses positive real part which is the growth rate determining the increment of the amplitude. Equation (30) is the condition of the presence of the growth rate and *u*_0,*cr*_ is related to shear modulus and Atwood number. Due to normally positive *G*_1_ and Atwood number in the range of (− 1, 1), the part in the radical sign of Eq. (30) is always greater than zero which means that a critical tangential velocity is always found to obtain a growth rate. Equation (30) indicates that fluid with smaller density needs to have a larger velocity in the tangential direction to overcome the effect of the mechanical property of solid to arouse the growth of the interface. If Eq. (30) is not satisfied, only imaginary part will be left in *n*_*e*_ which stands for the vibration frequency and the suppression of the growth.

In the plastic branch, the eigenvalue is

31 $$ \begin{aligned} n_{p} & = - M_{1} i \pm \sqrt { - M_{1}^{2} + M_{2} } \\ & = - \frac{{\rho_{2} ku_{0} }}{{\rho_{1} + \rho_{2} }}i \pm \frac{{k\sqrt {\rho_{1} \rho_{2} u_{0}^{2} } }}{{\rho_{1} + \rho_{2} }}, \\ \end{aligned} $$

where the index *p* denotes the plastic state of the solid. According to the form of Eq. (31), *ρ*_1_*ρ*_2_*u*_0_^2^ is always positive and *n*_*p*_ always has positive real part. The growth rate constantly exits in the plastic regime which leads to the possibility of amplitude growth, however, the growth is restrained in some situations. It is noticed that the growth rate is not regarding any information of the mechanical properties of solid and has the same form as the case of two ideal fluids with different densities. The model adopts the constitutive relation of perfect plasticity, of which the yield stress is constant and independent of the strain during deformation. So, the yield stress appears in the nonhomogeneous term in Eq. (23) and does not affect the growth rate but the solution of the amplitude. The suppression effects of the yield stress on amplitude growth will be shown in the analytical solutions.

### Analytical solutions for the perturbation amplitude

In order to solve Eq. (25) analytically, the following transformations are introduced

32a $$ x_{1} = \left[ {\Lambda \xi_{0} - \left( {\Lambda - M_{2} } \right)\xi } \right]e^{{iM_{1} t}} ,\quad \quad \xi \le \xi_{p} $$

32b $$ x_{2} = \left( {M_{2} \xi - {\rm X}} \right)e^{{iM_{1} t}} \;,\quad \quad \,\xi > \xi_{p} $$

and the two branches of Eq. (25) become

33a $$ \ddot{x}_{1} = \left[ { - M_{1}^{2} - \left( {\Lambda - M_{2} } \right)} \right]x_{1} \;,\quad \quad \xi \le \xi_{p} $$

33b $$ \ddot{x}_{2} = \left( { - M_{1}^{2} + M_{2} } \right)x_{2} ,\quad \quad \xi > \xi_{p} $$

which must follow the conditions of

34a $$ x_{1} \left( 0 \right) = M_{2} \xi_{0} \;,\quad \dot{x}_{1} \left( 0 \right) = iM_{1} M_{2} \xi_{0} \;, $$

34b $$ x_{1} \left( {t_{p} } \right) = x_{2} \left( {t_{p} } \right) = x_{p} \;, $$

34c $$ \dot{x}_{1} \left( {t_{p} } \right) = \dot{x}_{1p} ,\quad \dot{x}_{2} \left( {t_{p} } \right) = \dot{x}_{2p} , $$

where *t*_*p*_ is the time when the solid transients from elasticity to plasticity.

The solution of the amplitude can be derived by integrating the transformed motion equations. For the elastic branch, integration is conducted twice starting from Eq. (33a) with the initial conditions of Eq. (34a). Then, the transformation is inverted by substituting Eq. (32a) to get *ξ*(*t*) in the elastic state. Next, the same method is proceeded on Eq. (33b) for the plastic branch with the conditions of Eqs. (34b) and (34c) to derive *ξ*(*t*). Consequently, the solutions with complete analytical expressions for stable and unstable states are achieved.

Two stable solutions are

35 $$ \xi \left( t \right) = \frac{{\xi_{0} }}{{\Lambda - M_{2} }}\left\{ {\Lambda - \frac{1}{2}M_{2} e^{{ - iM_{1} t}} \left[ {\left( {1 + \frac{{M_{1} }}{{\sqrt {M_{1}^{2} + \left( {\Lambda - M_{2} } \right)} }}} \right)e^{{i\sqrt {M_{1}^{2} + \left( {\Lambda - M_{2} } \right)} t}} + \left( {1 - \frac{{M_{1} }}{{\sqrt {M_{1}^{2} + \left( {\Lambda - M_{2} } \right)} }}} \right)e^{{ - i\sqrt {M_{1}^{2} + \left( {\Lambda - M_{2} } \right)} t}} } \right]} \right\}\;\;, $$

for the purely elastic case below the transition point and

36 $$ \xi \left( t \right) = \left\{ \begin{gathered} \frac{{\xi_{0} }}{{\Lambda - M_{2} }}\left\{ {\Lambda - \frac{1}{2}M_{2} e^{{ - iM_{1} t}} \left[ {\left( {1 + \frac{{M_{1} }}{{\sqrt {M_{1}^{2} + \left( {\Lambda - M_{2} } \right)} }}} \right)e^{{i\sqrt {M_{1}^{2} + \left( {\Lambda - M_{2} } \right)} t}} + \left( {1 - \frac{{M_{1} }}{{\sqrt {M_{1}^{2} + \left( {\Lambda - M_{2} } \right)} }}} \right)e^{{ - i\sqrt {M_{1}^{2} + \left( {\Lambda - M_{2} } \right)} t}} } \right]} \right\}\;,\quad t \le t_{p} \hfill \\ \frac{1}{{M_{2} }}\left\{ {X + \frac{1}{2}e^{{ - iM_{1} t}} \left[ {\left( {x_{p} + \frac{{\dot{x}_{2p} }}{{\sqrt { - M_{1}^{2} + M_{2} } }}} \right)e^{{\left( {t - t_{p} } \right)\sqrt { - M_{1}^{2} + M_{2} } }} + \left( {x_{p} - \frac{{\dot{x}_{2p} }}{{\sqrt { - M_{1}^{2} + M_{2} } }}} \right)e^{{ - \left( {t - t_{p} } \right)\sqrt { - M_{1}^{2} + M_{2} } }} } \right]} \right\}\;,\quad t_{p} \le t \le t_{m} \hfill \\ \xi_{m} - \frac{{X - M_{2} \xi_{m} }}{{\Lambda - M_{2} }}\left\{ {1 - \frac{1}{2}e^{{ - iM_{1} \left( {t - t_{m} } \right)}} \left[ {\left( {1 + \frac{{M_{1} }}{{\sqrt {M_{1}^{2} + \left( {\Lambda - M_{2} } \right)} }}} \right)e^{{i\left( {t - t_{m} } \right)\sqrt {M_{1}^{2} + \left( {\Lambda - M_{2} } \right)} }} + \left( {1 - \frac{{M_{1} }}{{\sqrt {M_{1}^{2} + \left( {\Lambda - M_{2} } \right)} }}} \right)e^{{ - i\left( {t - t_{m} } \right)\sqrt {M_{1}^{2} + \left( {\Lambda - M_{2} } \right)} }} } \right]} \right\}\;,t \ge t_{m} \hfill \\ \end{gathered} \right. $$

for − *M*_1_^2^ − (Λ − *M*_2_) < 0 with EP transition. *t*_*p*_ is got by an implicit form by evaluating the first branch of Eq. (36) with *ξ*_*p*_, and *x*_*p*_ is given by evaluating Eq. (32a) at *t* = *t*_*p*_

37 $$ x_{p} = \left[ {\Lambda \xi_{0} - \left( {\Lambda - M_{2} } \right)\xi_{p} } \right]e^{{iM_{1} t_{p} }} .\quad \quad \xi \le \xi_{p} \; $$

Besides, it has

38 $$ \dot{x}_{2p} = \frac{1}{{\Lambda - M_{2} }}\left\{ {x_{p} M_{1} \Lambda i + M_{2} \sqrt {\left[ { - M_{1}^{2} - \left( {\Lambda - M_{2} } \right)} \right]x_{p}^{2} + \left( {\Lambda - M_{2} } \right)\left( {M_{2} \xi_{0} } \right)^{2} } } \right\}, $$

which is obtained by taking derivatives of Eqs. (32a) and (32b), then eliminating $\dot{\xi }$ from those two expressions and evaluating them at *t* = *t*_*p*_ with *x*_*p*_ and ${\dot{\text{x}}}_{1p}$ which is derived by integrating Eq. (33a) once and evaluating it at *t* = *t*_*p*_. *t*_*m*_ in Eq. (36) is the time of achieving the maximum amplitude *ξ*_*m*_ after plastic transition (*t*_*p*_ ≤ *t* ≤ *t*_*m*_) and the amplitude evolution goes back to perfectly elastic state after *t*_*m*_.

Two unstable solutions are, respectively,

39 $$ \xi \left( t \right) = \left\{ \begin{gathered} \frac{{\xi_{0} }}{{\Lambda - M_{2} }}\left\{ {\Lambda - \frac{1}{2}M_{2} e^{{ - iM_{1} t}} \left[ {\left( {1 + \frac{{M_{1} }}{{\sqrt {M_{1}^{2} + \left( {\Lambda - M_{2} } \right)} }}} \right)e^{{i\sqrt {M_{1}^{2} + \left( {\Lambda - M_{2} } \right)} t}} + \left( {1 - \frac{{M_{1} }}{{\sqrt {M_{1}^{2} + \left( {\Lambda - M_{2} } \right)} }}} \right)e^{{ - i\sqrt {M_{1}^{2} + \left( {\Lambda - M_{2} } \right)} t}} } \right]} \right\}\;,\quad t \le t_{p} \hfill \\ \frac{1}{{M_{2} }}\left\{ {X + \frac{1}{2}e^{{ - iM_{1} t}} \left[ {\left( {x_{p} + \frac{{\dot{x}_{2p} }}{{\sqrt { - M_{1}^{2} + M_{2} } }}} \right)e^{{\left( {t - t_{p} } \right)\sqrt { - M_{1}^{2} + M_{2} } }} + \left( {x_{p} - \frac{{\dot{x}_{2p} }}{{\sqrt { - M_{1}^{2} + M_{2} } }}} \right)e^{{ - \left( {t - t_{p} } \right)\sqrt { - M_{1}^{2} + M_{2} } }} } \right]} \right\}\;,\quad t \ge t_{p} \hfill \\ \end{gathered} \right. $$

for the case of − *M*_1_^2^ − (Λ − *M*_2_) < 0, and

40 $$ \xi \left( t \right) = \left\{ \begin{gathered} \frac{{\xi_{0} }}{{\Lambda - M_{2} }}\left\{ {\Lambda - \frac{1}{2}M_{2} e^{{ - iM_{1} t}} \left[ {\left( {1 + \frac{{iM_{1} }}{{\sqrt { - M_{1}^{2} - \left( {\Lambda - M_{2} } \right)} }}} \right)e^{{t\sqrt { - M_{1}^{2} - \left( {\Lambda - M_{2} } \right)} }} + \left( {1 - \frac{{iM_{1} }}{{\sqrt { - M_{1}^{2} - \left( {\Lambda - M_{2} } \right)} }}} \right)e^{{ - t\sqrt { - M_{1}^{2} - \left( {\Lambda - M_{2} } \right)} }} } \right]} \right\}\;,\quad t \le t_{p} \hfill \\ \frac{1}{{M_{2} }}\left\{ {X + \frac{1}{2}e^{{ - iM_{1} t}} \left[ {\left( {x_{p} + \frac{{\dot{x}_{2p} }}{{\sqrt { - M_{1}^{2} + M_{2} } }}} \right)e^{{\left( {t - t_{p} } \right)\sqrt { - M_{1}^{2} + M_{2} } }} + \left( {x_{p} - \frac{{\dot{x}_{2p} }}{{\sqrt { - M_{1}^{2} + M_{2} } }}} \right)e^{{ - \left( {t - t_{p} } \right)\sqrt { - M_{1}^{2} + M_{2} } }} } \right]} \right\}\;,\quad t \ge t_{p} \hfill \\ \end{gathered} \right. $$

for the case of − *M*_1_^2^ − (Λ − *M*_2_) > 0.

## Numerical simulations

Numerical simulations of KHI are inserted to state the reliability of the theoretical model. The perturbation growth factor which is the ratio of the amplitude history to the initial amplitude (i.e. *ξ*(*t*)/*ξ*_0_) is defined. The verification is implemented by comparing the growth factors obtained by model and simulations.

The numerical scheme is taken to be a 2D Lagrange method which has been adopted to simulate oblique shock wave impact problems and other hydrodynamic instability phenomena frequently^38,39^. Meshes are fixed to the material geometries at the initial moment and transform with the motion of the materials in the Lagrange method which can capture the material interface clearly. The framework of the Lagrange method is illustrated in the Ref.^41^ in details and the finite element method will be not presented specifically here.

**Figure 2:**
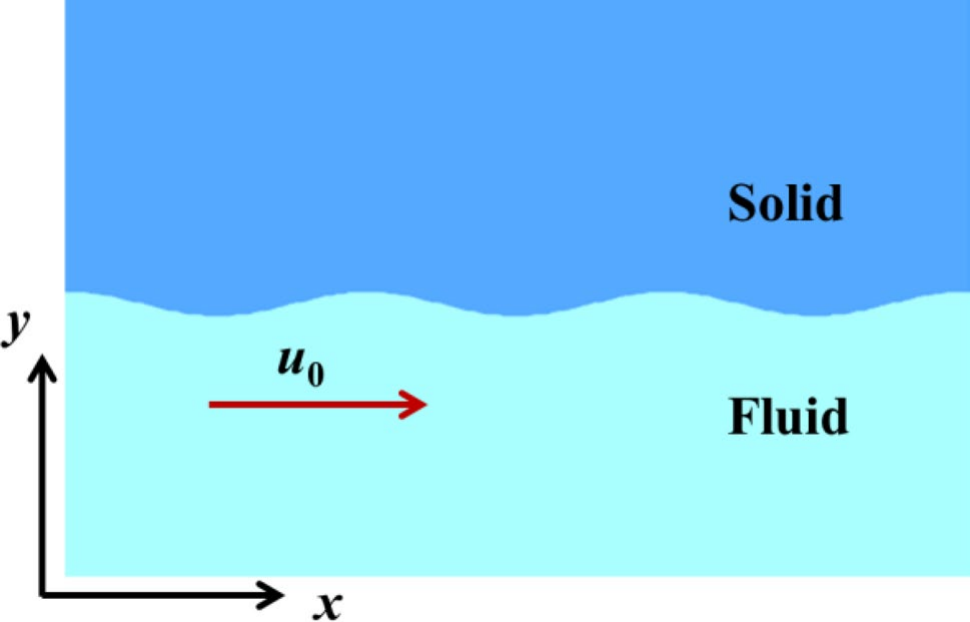
The material interface at initial moment.

Two plates are considered in Cartesian planar coordinates in the simulations. The initial conditions are shown in Fig. 2. The initial amplitude (2*ξ*_0_) which is the distance from wave crest to trough in the *y* direction is 20 μm and the wavelength is 250 μm which are representative characteristic scales in oblique impact^25,39^. Both of the thicknesses of two plates are set to be 2 mm to guarantee *kh*_1_ ≫ 1 and *kh*_2_ ≫ 1, where *h*_1_ and *h*_2_ are the thicknesses for solid and fluid respectively, to ensure the developments of the instabilities behave like half mediums. The lengths of two plates in the *x* direction contain twenty wavelengths to diminish the effect of lateral extents. Square meshes with the size of 2.5 μm are distributed near the interface at the initial moment. The solid plate is quiescent and the tangential velocity of the fluid plate is set to be *u*_0_ at the initial time.

The properties of solid and fluid are simulated with a Mie-Grüneisen EOS with a coefficient Г = *ρ*_0_Г_0_/*ρ*, where Г_0_ is a parameter characteristic of a material and *ρ*_0_ is the density under normal conditions. The shock velocity *v*_*s*_ is assumed to linearly varies with the particle velocity *v*_*p*_, i.e. *v*_*s*_ = *c*_0_ + *sv*_*p*_, where *c*_0_ and *s* are characteristic constants of the material. The solid material is set to be copper which appears in the oblique impact experiment^16^, as the fluid is set to be water. The parameters of EOS for solid and fluid are listed in Table 1. In the simulations, a value of 10*c*_0_ is taken to the original *c*_0_ in Table 1 to ensure incompressibility. Besides, a perfectly elastic and rigid plastic model is adopted to characterize the copper. The values of the shear modulus *G*_1_ and the yield strength *Y* are taken as constants. The specific values of *G*_1_ and *Y* are also given in Table 1.

**Table 1:** Parameters in simulations for solid and fluid^42–44^.

	*ρ* (g/cm^3^)	Г_0_	*c*_0_ (cm/μs)	*s*	*G*_1_ (GPa)	*Y* (MPa)
Copper	8.9	2.02	3.94	1.49	39.38	500
Water	1.0–13.0	0.4934	1.48	2.56	–	–

Although the verification of the Lagrange code has been applied in Ref.^41^, the situation in Ref.^41^ is not completely consistent with that we settle here. Hence, the evolution of the amplitude in KHI by numerical simulation is compared with the classical theoretical formulation of two ideal fluids to perform the credibility by considering lack of the experiment at the same condition of the theoretical analysis. As regarding the simplest case of identical ideal fluids, the amplitude by classical theory varies exponentially with time corresponding to the situation of *A*_*T*_ = 0 in the linear growth stage^38,39,45^

41 $$ \xi \left( t \right) \sim \xi_{0} \cosh \left( {\frac{{ku_{0} }}{2}t} \right). $$

**Figure 3:**
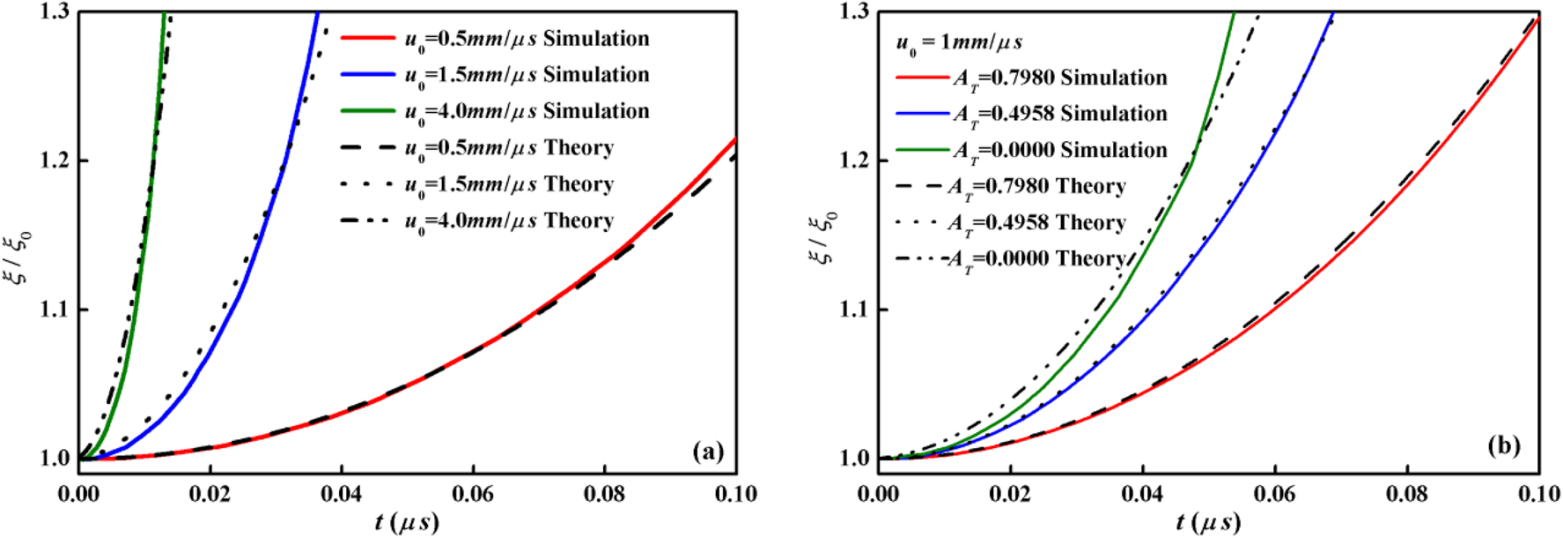
Growth factors versus time by numerical simulations and classical theory of KHI between two ideal fluids: (**a**) *u*_0_ = 0.5, 1.5, 4.0 mm/μs as *A*_*T*_ = 0, (**b**) *A*_*T*_ = 0.7980, 0.4958, 0.0 as *u*_0_ = 1.0 mm/μs.

Three comparisons at different *u*_0_, i.e. 0.5, 1.5, 4.0 mm/μs, are practiced in Fig. 3a. The growth factors calculated by numerical code and theoretical formulation in Eq. (41) have good agreements in the early growth phase. The divergences in the late time are detected due to the departure of the linear growth stage. Furthermore, the amplitude for two ideal fluids with distinct densities by theory is

42 $$ \xi \left( t \right) \sim \xi_{0} \cosh \left( {\sqrt {\frac{{\rho_{1} \rho_{2} }}{{\left( {\rho_{1} + \rho_{2} } \right)^{2} }}} ku_{0} t} \right). $$

Figure 3b plots the growth factors by numerical method and Eq. (42) at *u*_0_ = 1 mm/μs at different Atwood number. *ρ*_1_ is set to be 8.9 g/cm^3^ and *ρ*_2_ is altered to obtain different Atwood numbers with values of 1.0, 3.0 and 8.9 g/cm^3^ respectively. Good comparisons in the early stage are shown again.

**Figure 4:**
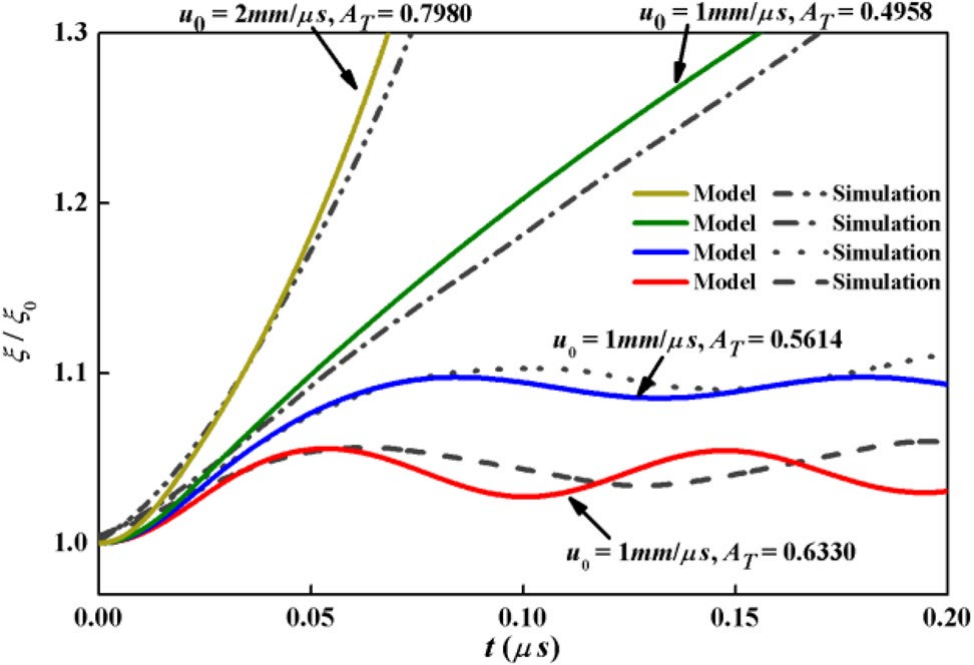
Growth factors versus time by model and simulation of KHI between perfectly EP solid and ideal fluid.

After perform the credibility of the Lagrange code, the growth factors achieved by the analytical solutions of the amplitude are plotted in company with the growth factors by simulations in Fig. 4 where two stable solutions and two unstable solutions are shown. The copper is stationary at the initial moment and the water with relative tangential velocity is set below. The material properties of solid and fluid in Table1 are also adopted. The conditions, including initial amplitude, wavelength, tangential velocities and material parameters are identical in the theoretical model and simulations. The stable cases correspond to *u*_0_ = 1.0 mm/μs, *ρ*_2_ = 2.0 g/cm^3^ and *u*_0_ = 1.0 mm/μs, *ρ*_2_ = 2.5 g/cm^3^, as the unstable cases correspond to *u*_0_ = 1.0 mm/μs, *ρ*_2_ = 3.0 g/cm^3^ and *u*_0_ = 2.0 mm/μs, *ρ*_2_ = 1.0 g/cm^3^. The growth factors by model are basically consistent with those by simulations. Main characteristics of growth factors are obtained by the model, such as the oscillations in the stable case and the growths as the interface is unstable. The limited differences may because the discontinuity of tangential velocities near the interface always maintains in the model whereas a continuous profile of the tangential velocity is gradually established in the simulation.

## Results and discussion

**Figure 5:**
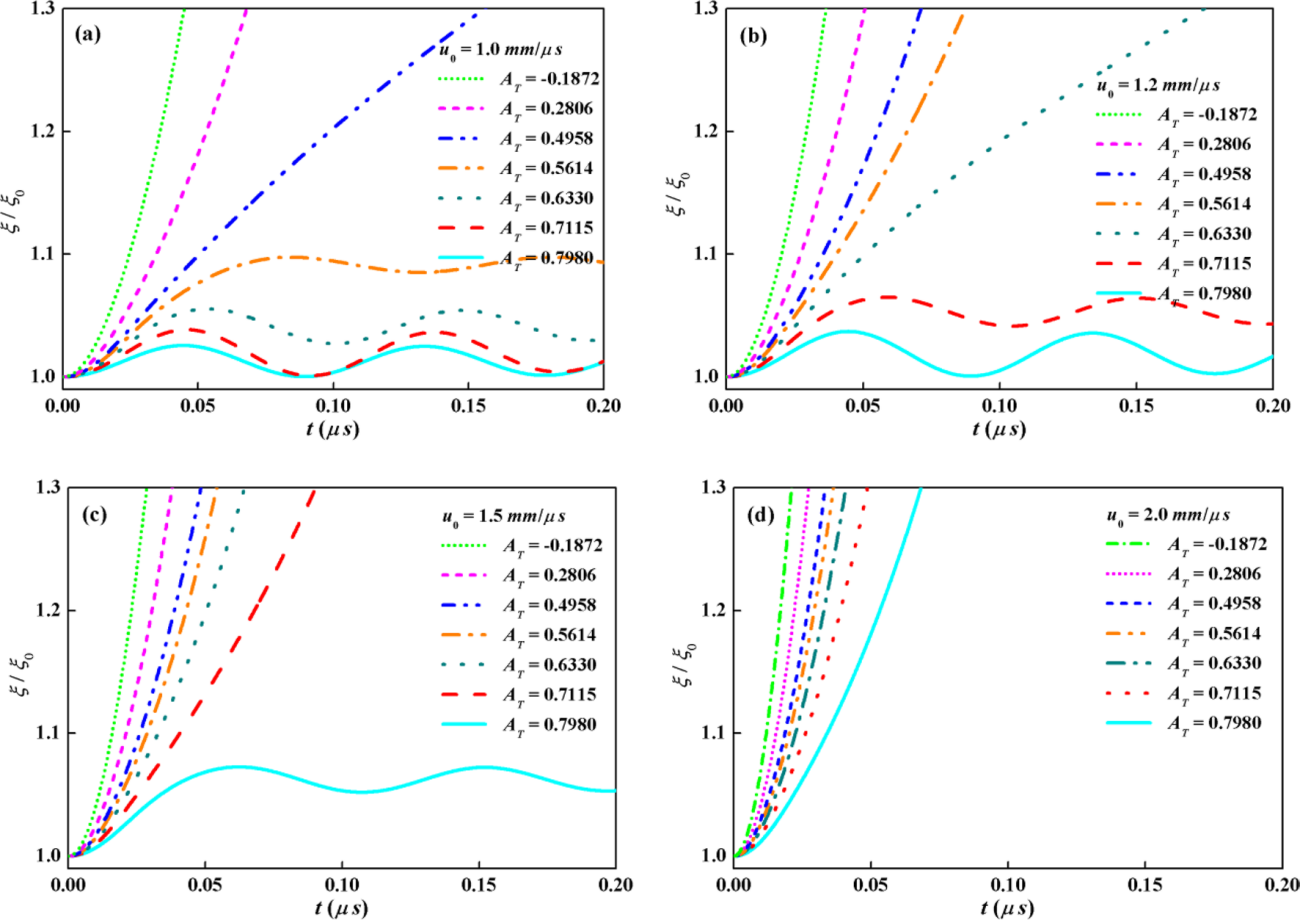
Growth factors vs time with *A*_*T*_ = 0.7980, 0.7115, 0.6330, 0.5614, 0.4958, 0.2806 and − 0.1872 at *u*_0_ by analytical model: (**a**) *u*_0_ = 1 mm/μs, (**b**) *u*_0_ = 1.2 mm/μs, (**c**) *u*_0_ = 1.5 mm/μs, (**d**) *u*_0_ = 2.0 mm/μs.

Characteristics of the evolution of the perturbation amplitude and effects of Atwood number based on the theoretical model are illustrated particularly in this section. The developments of KHI between EP solid and ideal fluid at *u*_0_ = 1.0 mm/μs with different *A*_*T*_ are plots in Fig. 5a. Material interface is the same as that in Fig. 2 i.e. *ξ*_0_ = 10 μm and *λ* = 250 μm. The solid plate is also set as copper with the mechanical parameters listed in Table 1, i.e. *ρ*_1_ = 8.9 g/cm^3^, *G*_1_ = 39.38 GPa, *Y* = 0.5 GPa. The values of *ρ*_2_ are taken to be 1.0, 1.5, 2.0, 2.5, 3.0, 5.0 and 13.0 g/cm^3^ to get the values of *A*_*T*_ to be 0.7980, 0.7115, 0.6330, 0.5614, 0.4958, 0.2806 and -0.1872 respectively. When *A*_*T*_ equals 0.7980 or 0.7115, the interface is stable in the purely elastic stage. According to Eq. (30), *u*_0_ is smaller than the critical velocity. The eigenvalue in Eq. (29) has only the imaginary part which means an oscillation frequency. The amplitudes follow the solution in Eq. (35). The growth factors vibrate around small values and the amplitudes are always below the plastic transition point *ξ*_*p*_ in Eq. (21). The growth of the interface is controlled by the mechanical properties *G*_1_. The amplitude increases slightly as *A*_*T*_ decreases from 0.7980 to 0.7115. In the cases of *A*_*T*_ = 0.6330 and 0.5614, *u*_0_ is still smaller than the critical velocity and the amplitudes perform the vibration behaviors. Yet, the vibration is strong enough to achieve the EP transition point. After solid transforms from elastic state into plastic state, the amplitudes grow to maximum value *ξ*_*m*_ at time *t*_*m*_ by the suppression of material yield stress, even though there is always a growth rate in the plastic regime as shown in Eq. (31). Since arriving at the maximum value, the evolutions of the amplitudes go back to the elastic state to oscillate near the maximum amplitude based on Eq. (36). For the cases of *A*_*T*_ = 0.4958, 0.2806 and -0.1872, the interface also vibrates from elastic state to plastic state after EP transition point. However, the growth of amplitude cannot be suppressed by the yield stress. The growth factors keep the trends of increments according to the solution in Eq. (39). The KHI system is unstable under these situations. The slope of the growth factor of *A*_*T*_ = 0.4958 is obviously less than that of *A*_*T*_ = 0.2806 and − 0.1872. The speed of the growth with *A*_*T*_ = − 0.1872 is the most rapid in the seven *A*_*T*_ cases in Fig. 5a.

Furthermore, the tangential velocity *u*_0_ is enhanced and other parameters are fixed the same as the conditions in Fig. 5a. Three more values of *u*_0_ = 1.2, 1.5 and 2.0 mm/μs are exhibited in Fig. 5b,c and d respectively. Under disparate velocities, the effects of Atwood number on the developments of the perturbation display distinct features. As the velocity varying from 1.0 mm/μs to 1.2 mm/μs, the development of the growth factor of case *A*_*T*_ = 0.7980 is still stable in the purely elastic stage just with the increase of the amplitude. For the case of *A*_*T*_ = 0.7115, the characteristics of the growth factor convert from the elastic vibration in Fig. 5a to the plastic stable state in Fig. 5b. The suppression mode of the growth is controlled by *Y*, not by *G*_1_. As noticed in the cases of *A*_*T*_ = 0.6330, 0.5614, 0.4958, 0.2806 and − 0.1872, every interface is unstable. When the velocity arrives at 1.5 mm/μs in Fig. 5c, except for the largest one of the seven *A*_*T*_, the systems of KHI between EP solids and ideal fluids grow rapidly to be unstable. The stable one follows the solution in Eq. (36) but not the purely elastic one in Eq. (35). For an even higher velocity *u*_0_ = 2.0 mm/μs, none stable interface is detected in the Fig. 5d.

**Figure 6:**
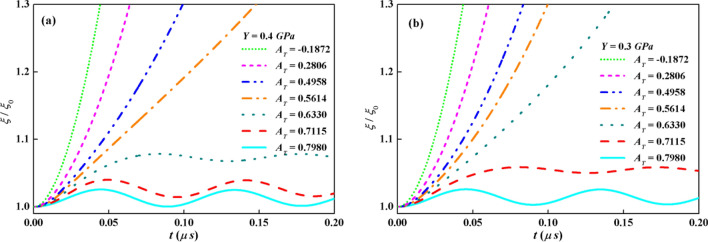
Growth factors vs time with *A*_*T*_ = 0.7980, 0.7115, 0.6330, 0.5614, 0.4958, 0.2806 and − 0.1872 at different *Y* by analytical model: (**a**) *Y* = 0.4 GPa, (**b**) *Y* = 0.3 GPa.

Figure 6a and b plot the growth factors with the same parameters in Fig. 5a except for *Y* = 0.4 GPa and *Y* = 0.3 GPa. A purely elastic solution as *A*_*T*_ = 0.7980 are observed in Fig. 6a as *Y* = 0.4 GPa. The growth factor vibrates beneath *ξ*_*p*_ and the interface maintains the stable state. Two stable examples with EP transition as *A*_*T*_ = 0.7115 and 0.6330 are also given in Fig. 6a. The amplitudes remain elastic oscillation after growing into the plastic stage. In the latter cases as *A*_*T*_ = 0.5614, 0.4958, 0.2806 and -0.1872, the perturbation amplitudes are unstable and increase with different slopes. As expected, the amplitude with the largest *ρ*_2_ i.e. *A*_*T*_ = -0.1872, develops fastest. By decreasing the yield stress to 0.3GPa, as shown in Fig. 6b, each of the stable cases as *A*_*T*_ = 0.7980 and 0.7115 follows Eq. (36) with EP transition. Purely elastic solution is not found as *Y* = 0.3 GPa in the seven *A*_*T*_ cases. For the other five *A*_*T*_, the systems of KHI are unstable after the amplitudes have grown sufficiently to the plastic regime. As shown in Fig. 6a and b, the suppression effects of yield strength diminish as reducing *Y*.

**Figure 7:**
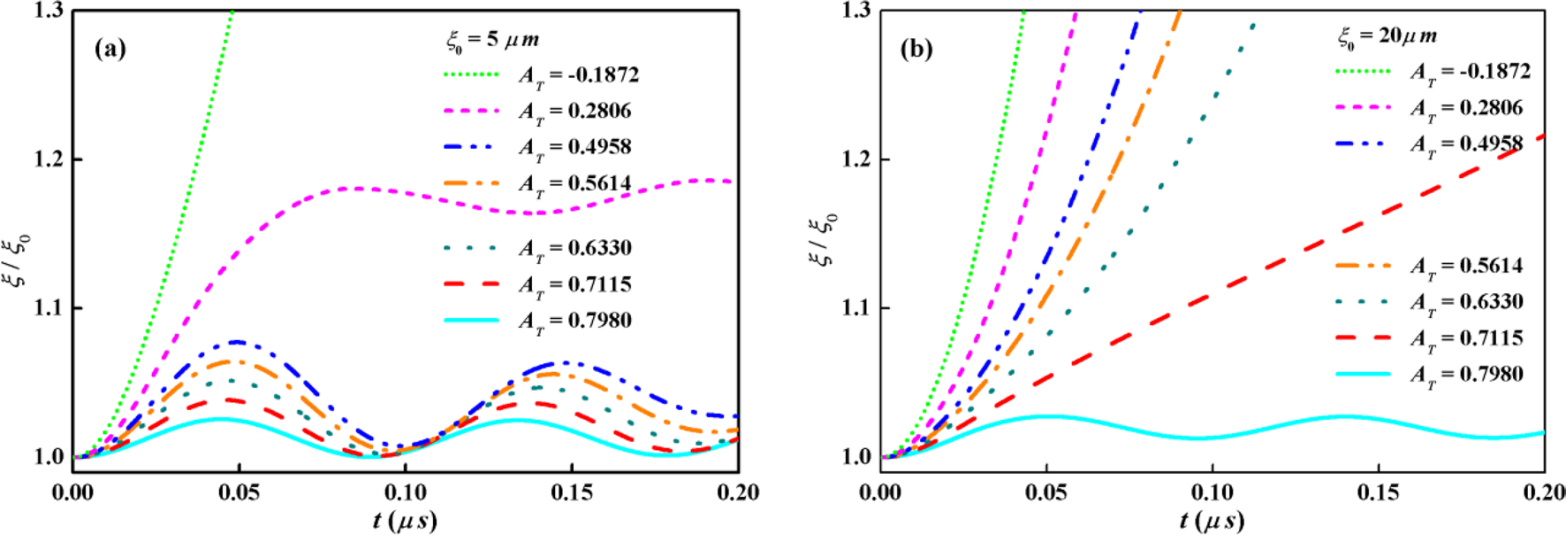
Growth factors vs time with *A*_*T*_ = 0.7980, 0.7115, 0.6330, 0.5614, 0.4958, 0.2806 and − 0.1872 with different *ξ*_0_ by analytical model: (**a**) *ξ*_0_ = 5 μm, (**b**) *ξ*_0_ = 20 μm.

Another aspect is discussed to perform some features when the initial amplitude varies. Compared with conditions in Fig. 5a, *ξ*_0_ is changed to be 5 μm and 20 μm. The growth factors for the two values of *ξ*_0_ are presented in Fig. 7a and b respectively. In Fig. 7a, cases of *A*_*T*_ = 0.7980, 0.7115, 0.6330, 0.5614 and 0.4958 as *ξ*_0_ = 5 μm are stable in the purely elastic regime under the EP transition point *ξ*_*p*_. As the expression in Eq. (21), *ξ*_*p*_ is determined by *ξ*_0_ and a term with regard to *k*, *G*_1_ and *Y*. The decrement of *ξ*_0_ diminishes *ξ*_*p*_ apparently but enhances the ratio of *ξ*_*p*_ and *ξ*_0_ because *k*, *G*_1_ and *Y* are fixed. The amplitude of the elastic oscillation increases as *A*_*T*_ decreases. In the case of *A*_*T*_ = 0.2806, the interface experiences EP transition and then oscillates elastically. The growth factor losses stability after EP transition as *A*_*T*_ = − 0.1872. Afterwards, to investigate the amplitude evolution of the initial amplitude with larger value, *ξ*_0_ is raised to 20 μm and the growth factors with the upper seven *A*_*T*_ are plotted in Fig. 7b. *ξ*_*p*_/*ξ*_0_ decreases and the case of *A*_*T*_ = 0.7980 follows the stable solution with EP transition. The other six KHI systems are unstable.

The above results indicate that the evolution of the perturbation interface in KHI system between EP solid and ideal fluid is influenced by *A*_*T*_. The developments of the amplitude perform disparate behaviors as varying *A*_*T*_ when other parameters are invariable. With decreasing *A*_*T*_, the system changes from stable state to unstable state. The purely elastic stable state is controlled by *G*_1_, as well as the stable state with plastic transition is affected by the EP properties of solid. The vibration of the amplitude is detected in both of the stable situations. As the density of fluid increases, the amplitude grows to lose stability. For the cases of *A*_*T*_ = 0.7980 and 0.7115 in the three groups of different *u*_0_, *Y* and *ξ*_0_, the evident distinctions of the evolution of the amplitude are explored. The characteristics of the amplitude motion are prominently governed by the EP constitutive relation to present various trends of stable and unstable states. But, for the case of *A*_*T*_ = − 0.1872, the amplitude increases in each situation and the suppression effects of the EP properties of solid seem to be invalid. Therefore, an inspiration is brought that it is necessary to pay attention on the suppression effects of EP properties which may influence the formation of the wavy morphology while plates with large *A*_*T*_ are suffering obliquely impact.

## Conclusion

The evolution of the amplitude and the Atwood number effects in KHI system between EP solid and ideal fluid is discussed by developing an approximate theoretical model which is convenient to derive the analytical solution of the perturbation amplitude in present study.

Based on the assumptions of incompressible and irrotational flow field, the velocities are expressed by the velocity potentials. Arbitrary densities are contained in the momentum equations which are integrated to obtain pressures. Based on the continuity of normal velocities and force equilibrium with non-linear EP mechanical properties of solid at interface, the governing equations for the motion of the amplitude can be achieved. It is possible to solve the motion equations by some transformations and integrations to obtain completely analytical solutions which have two stable and two unstable expressions. The understanding of KHI can be quantitatively analyzed when the surface of EP solid is flowed over by ideal fluid with constant tangential velocities.

The verification is actualized by comparing the distributions of the growth factors by 2D Lagrange simulations and theoretical model. The credibility of the model is performed by the consistent results for both stable and unstable cases. The evolution of the amplitude can be described by the theoretical model.

Several groups of the growth factors by the model are presented in the article. One stable evolution of the amplitude in the purely elastic stage exhibits the behavior of vibration below the EP transition point under the suppression of solid shear modulus which controls the growth rate. By mentioning the other stable solution with EP transition, the amplitude elastically vibrates sufficiently to transform from elastic to plastic regime, and remains oscillating elastically after it grows to a maximum point in the plastic regime. The yield strength governs the motion of the amplitude after plastic transition but not by preventing the growth rate. For the unstable case, the system losses stability after EP transition and the amplitude keeps increasing. As Atwood number decreasing, the perturbed interface deviates from stable to unstable situations. The controlling effects of EP properties play a dominant role on the development processes with large *A*_*T*_. When two different plates with large *A*_*T*_ undergo oblique impact, the EP mechanical properties of the solid may need to be considered carefully in the analysis of the evolution of KHI. The relevant research will be investigated particularly in future work.
